# Multiomics reveals postherbivory tea leaves restructure endophytic microbiota to resist disease and boost saccharide metabolism to promote growth

**DOI:** 10.1093/hr/uhag338

**Published:** 2026-08-04

**Authors:** Yishuai Yang, Zihan Shi, Yulian Deng, Chengwen Shen, Linghong Zhou, Ye Deng, Lin Tan, Zhonghua Liu, Qiulong Hu

**Affiliations:** College of Horticulture, Hunan Agricultural University, Changsha, Hunan 410128, China; College of Plant Protection, Hunan Agricultural University, Changsha, Hunan 410128, China; National Research Center of Engineering and Technology for Utilization of Botanical Functional Ingredients & Co-Innovation Center of Education Ministry for Utilization of Botanical Functional Ingredients, Hunan Agricultural University, Changsha 410128, China; Key Laboratory of Tea Science of Ministry of Education, Yuelushan Laboratory, Hunan Agricultural University, Changsha 410128, China; State Key Laboratory of Tea Plant Germplasm Innovation and Resource Utilization, Hunan Agricultural University, Changsha 410128, China; College of Horticulture, Hunan Agricultural University, Changsha, Hunan 410128, China; College of Plant Protection, Hunan Agricultural University, Changsha, Hunan 410128, China; National Research Center of Engineering and Technology for Utilization of Botanical Functional Ingredients & Co-Innovation Center of Education Ministry for Utilization of Botanical Functional Ingredients, Hunan Agricultural University, Changsha 410128, China; Key Laboratory of Tea Science of Ministry of Education, Yuelushan Laboratory, Hunan Agricultural University, Changsha 410128, China; State Key Laboratory of Tea Plant Germplasm Innovation and Resource Utilization, Hunan Agricultural University, Changsha 410128, China; College of Horticulture, Hunan Agricultural University, Changsha, Hunan 410128, China; National Research Center of Engineering and Technology for Utilization of Botanical Functional Ingredients & Co-Innovation Center of Education Ministry for Utilization of Botanical Functional Ingredients, Hunan Agricultural University, Changsha 410128, China; Key Laboratory of Tea Science of Ministry of Education, Yuelushan Laboratory, Hunan Agricultural University, Changsha 410128, China; State Key Laboratory of Tea Plant Germplasm Innovation and Resource Utilization, Hunan Agricultural University, Changsha 410128, China; College of Horticulture, Hunan Agricultural University, Changsha, Hunan 410128, China; National Research Center of Engineering and Technology for Utilization of Botanical Functional Ingredients & Co-Innovation Center of Education Ministry for Utilization of Botanical Functional Ingredients, Hunan Agricultural University, Changsha 410128, China; Key Laboratory of Tea Science of Ministry of Education, Yuelushan Laboratory, Hunan Agricultural University, Changsha 410128, China; State Key Laboratory of Tea Plant Germplasm Innovation and Resource Utilization, Hunan Agricultural University, Changsha 410128, China; Technology Center, Chenzhou Agricultural Science Research Institute, Chenzhou 423001, China; CAS Key Laboratory for Environmental Biotechnology, Research Center for Eco-Environmental Sciences, Chinese Academy of Sciences (CAS), Beijing, China; College of Plant Protection, Hunan Agricultural University, Changsha, Hunan 410128, China; National Research Center of Engineering and Technology for Utilization of Botanical Functional Ingredients & Co-Innovation Center of Education Ministry for Utilization of Botanical Functional Ingredients, Hunan Agricultural University, Changsha 410128, China; Key Laboratory of Tea Science of Ministry of Education, Yuelushan Laboratory, Hunan Agricultural University, Changsha 410128, China; State Key Laboratory of Tea Plant Germplasm Innovation and Resource Utilization, Hunan Agricultural University, Changsha 410128, China; College of Horticulture, Hunan Agricultural University, Changsha, Hunan 410128, China; National Research Center of Engineering and Technology for Utilization of Botanical Functional Ingredients & Co-Innovation Center of Education Ministry for Utilization of Botanical Functional Ingredients, Hunan Agricultural University, Changsha 410128, China; Key Laboratory of Tea Science of Ministry of Education, Yuelushan Laboratory, Hunan Agricultural University, Changsha 410128, China; State Key Laboratory of Tea Plant Germplasm Innovation and Resource Utilization, Hunan Agricultural University, Changsha 410128, China; College of Horticulture, Hunan Agricultural University, Changsha, Hunan 410128, China; National Research Center of Engineering and Technology for Utilization of Botanical Functional Ingredients & Co-Innovation Center of Education Ministry for Utilization of Botanical Functional Ingredients, Hunan Agricultural University, Changsha 410128, China; Key Laboratory of Tea Science of Ministry of Education, Yuelushan Laboratory, Hunan Agricultural University, Changsha 410128, China; State Key Laboratory of Tea Plant Germplasm Innovation and Resource Utilization, Hunan Agricultural University, Changsha 410128, China

## Abstract

The induced capacity of plant growth-defense trade-offs under biotic stress is increasingly recognized. While many studies focus primarily on plants’ defense activation stage during insect attacks, limited attention has been given to poststress recovery. Herein, leaves from normal and postherbivory tea plants were collected during the compensation stage, and their microbiomes, metabolomes, and transcriptomes were used to dissect the mechanisms underlying growth-defense trade-offs. The results revealed that apart from altering the microbial community diversity, insect herbivory significantly enriched leaf-associated pathogens, particularly *Alternaria spp.* Network analysis and in-situ separation jointly revealed the role of *Sphingomonas aquatilis* in resisting pathogen invasion. Meanwhile, the restructured microbiota exhibited stronger network stability, indicating the enhancement of pathogen resistance among the endophytic community. Moreover, integrated transcriptomic and metabolomic analysis revealed that genes such as GSGT1, GolS4, and α-gal may regulate synthesis and degradation of growth-promoting and defense metabolites. The downregulated flavonoids were defense compounds against pathogens and the upregulated saccharides were plant growth-promoting compounds, which were verified in subsequent tests. Overall, tea plants compensate for reduced defense through restructuring of the endophytic microbiota, and prioritize growth over defense through metabolic resetting after insect herbivory. This study also revealed new biocontrol and growth-promoting resources for tea plants.

## Introduction

As an important economic crop, tea plants (*Camellia sinensis*) often face unfavorable environmental conditions across their life cycle, especially damage from insects [1], and necessitate iterative recovery from recurring insect infestations. Theoretical frameworks posit that insect herbivory causes tea plant leaf wounds, which are conducive to pathogen invasion and have a negative impact on tea yield [2, 3]. However, field observations in commercial plantations reveal a counterintuitive phenomenon: tea plants manifest exceptional ecological adaptability and enable sustained productivity maintenance without the use of pesticides [4]. The tea plant’s sophisticated adaptive traits establish it as a premier biological model [5], which enables precise dissection of the autogenous defense and recovery resilience mechanisms of plants. What mechanisms enable tea plants to activate defense responses against pathogenic invasions while coordinating growth recovery processes following insect herbivory? This scientific issue merits in-depth and systematic research. Meanwhile, it is also essential to fully explore defense-related and growth-promoting resources for tea plants during the postherbivory recovery stage during severe pests outbreaks.

During the long-term tripartite coexistence of plants, insects, and microorganisms, all three groups have undergone co-evolution, driving the emergence of complex interaction mechanisms that directly influence plant growth and productivity [6, 7]. Insect herbivory can penetrate this community’s defenses and suppress plant immunity by introducing and transmitting pathogenic microorganisms, ultimately leading to plant disease outbreaks [8, 9]. Previous research has advanced the understanding of short-term induced plant defenses and acute stress responses to insect attacks, which typically occur in a few minutes or even seconds [10]. However, critical gaps persist in understanding the growth-defense trade-offs during the recovery stage of plants subjected to biological stresses (e.g. insect attack or pathogen invasion).

The mechanism of growth-defense trade-offs means that when normally growing plants are threatened by pests and diseases, they improve their defense capabilities to sustain their growth through resource allocation, metabolic reset, hormonal modulation, and gene regulation [11, 12]. Metabolites are the major resource playing important roles in plant defense and are broadly classified as constitutive defense metabolites and inducible defense compounds [13]. Compared to constitutive defense, inducible defense is more energetically economical and plays a crucial role in plant defense [14]. Plants can produce various defense metabolites, increase reactive oxygen species levels, and synthesize phytohormones to directly or indirectly defend against pest attacks after being induced by their herbivory [15, 16]. In plants, the activation of defense mechanisms is often accompanied by suppressed growth, as resource allocation prioritizes stress responses over developmental processes, as described by the growth-defense trade-offs theory [17]. Despite its pivotal role in evolutionary biology and plant physiology, current research mainly focuses on defense activation mechanisms, while poststress recovery mechanisms remain severely under-characterized.

In addition to regulating metabolic reprogramming, microorganisms, particularly endophytes, play a critical role in plant disease resistance [18, 19]. Numerous studies have confirmed that endophytes restrict pathogen colonization through multiple pathways: synthesis of antimicrobial metabolites [20], competition for niches and nutrients [21, 22], and induction of plant systemic resistance [19]. Plants actively assemble beneficial endophyte communities to resist pathogens and alleviate damage caused by persistent insect herbivory [23, 24]. These microbe-driven defenses are therefore integral to the plant immune system. Insect herbivory degrades plant structural defenses through mechanical disruption of epidermal tissues. These insect-induced wounds establish critical entry points that act as microbial colonization gateways, facilitating the establishment of infection sites for microconidial invasion [2]. Unlike the vector-mediated pathogen transmission confined to specific host-plant interactions, this insect-induced wound constitutes a ubiquitous biotic stressor that persists throughout all developmental stages of plant life cycles. By reshaping the microbial composition, plants can enhance their resistance to pathogen invasion and promote healthy growth [25, 26]. This adaptive response is crucial for maintaining plant health under biotic stress conditions. However, current plant-microbe interaction paradigms inadequately delineate the ecological impacts of insect herbivory-induced microbial community reorganization, particularly the reconstruction of key endophytic communities that maintain plant-microbe symbiosis homeostasis during the recovery growth stage of the plant after insect herbivory. This knowledge gap hinders our ability to understand how nonvector insects indirectly shape plant autogenous resilience strategy through restructuring of the endophytic microbiota.

**Figure 1:**
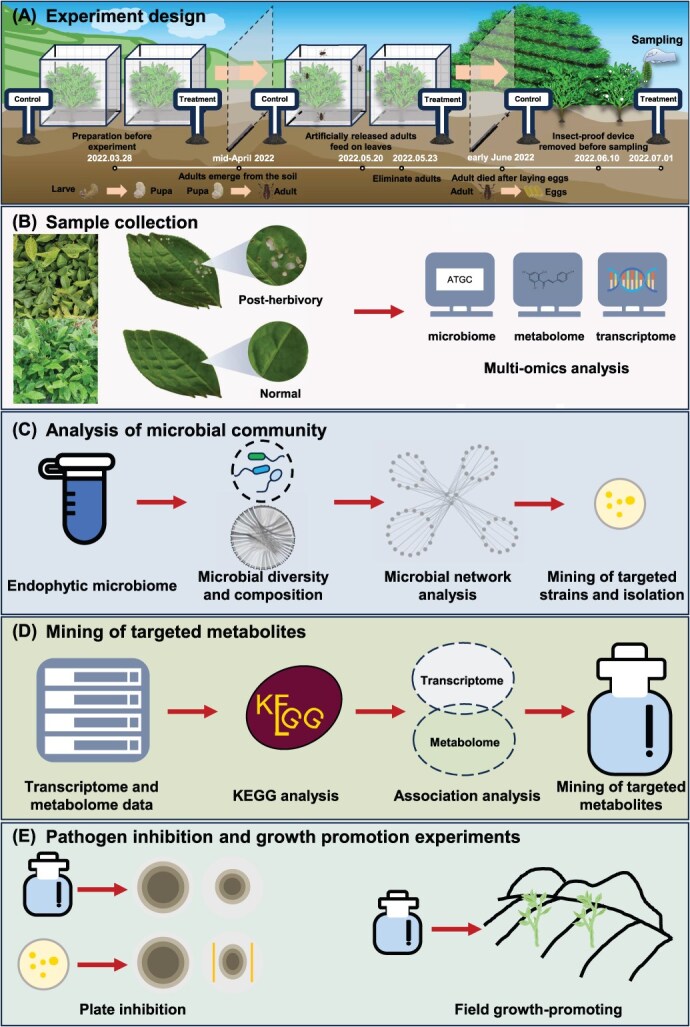
Overall research ideas and methods. (A) A controlled field experiment was designed for sample collection. Two experimental groups were set up: the nonherbivory-treated control group and the herbivory-treated group (exposed to *B. melanopus*). (B) After pretreatment, the samples were extracted and sequenced. (C) Targeted strains were mined based on microbial network analysis, and then isolated through in situ isolation. (D) Integrated analysis of KEGG pathways from metabolome and transcriptome was conducted to mine targeted metabolites. (E) The function verification of mined strains and metabolites was carried out.

Multiomics analysis integrating microbiome, transcriptomic, and metabolomic data is crucial for comprehensively understanding plant biological processes [27]. Understanding the interactions among microorganisms, metabolites, and gene transcription during a plant’s recovery growth stage after insect herbivory is crucial for deciphering the growth-defense mechanisms. Here, we hypothesized that plants prioritize growth over defense during the postherbivory recovery stage through microbiome restructuring, metabolic resetting, and transcriptome reprogramming, and aimed to elucidate the growth-defense trade-offs during this critical regeneration phase. To investigate this hypothesis, we targeted the specialized interaction between tea plants and *Basilepta melanopus* (leaf beetle) within their co-evolved native ecosystems, thus providing an ideal system for studying persistent plant growth-defense mechanisms. Restructuring of endophytic microbial communities and the differential expression of metabolites and genes in leaves from plants that experienced insect herbivory or were normal (control) were examined using amplicon sequencing, metabolomics, and transcriptomics. The growth recovery-related metabolites and defense-related beneficial microbiota were analyzed, and their roles in promoting plant growth or inhibiting pathogens were verified and evaluated. Finally, the mechanisms underlying the plant recovery process after insect herbivory were elucidated based on the potential roles of crucial genes and the verified functions of the beneficial microbes and metabolites (Fig. 1). This study expands the theoretical understanding of plant growth-defense trade-offs under pest stress and provides a foundation for the development of novel products enhancing disease resistance and promoting growth of tea plants.

## Results

### Alterations in the phyllosphere endophyte microbiome postherbivory

**Figure 2:**
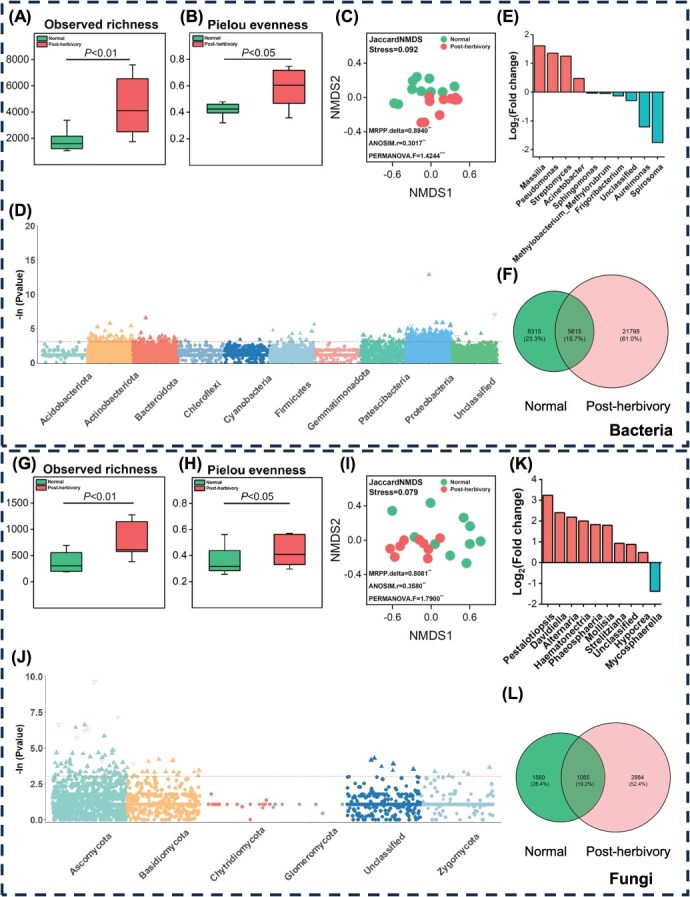
Postherbivory and normal endogenous microbiotas for differential bacterial and fungal diversity, community structure, and taxonomy. Alpha-diversity based on observed richness and Pielou’s evenness for bacterial (A and B) and fungal (G and H) communities, assessed by Student’s *t*-test. Nonmetric multidimensional scaling analysis with Jaccard metrics was conducted to characterize the beta diversity and analyzed including MRPP, ANOSIM, and PERMANOVA to test for bacterial (C) and fungal (I) differences. Manhattan plots showing differential OTUs in postherbivory samples for bacterial (D) and fungal (J) microbiota. Filled and empty triangles denote enriched and depleted OTUs, respectively (*P* < 0.05). Each symbol represents one OTU. Besides, all circles indicate OTUs without significance between the two groups. The enriched or depleted bacterial (E) and fungal (K) genera of the postherbivory samples (the top 10 relative abundance genera). Data bars represent the log_2_ fold-change (postherbivory/normal ratios). Venn diagram depicting the number of OTUs identified in bacterial (F) and fungal (L) microbiota from the postherbivory and normal samples.

According to the diversity indices of richness and evenness, the postherbivory leaf samples exhibited greater microbial richness and evenness for both endophytic bacteria and fungi (Student’s *t*-test, *P* < 0.05) (Fig. 2A, B, G, and  H). A clear separation between microbial communities was observed in the nonmetric multidimensional scaling analysis, indicating a significant difference in the community structure of leaf endophytic microbiomes between postherbivory and normal plants (Fig. 2C and I).

After filtering to remove nontarget sequences, a total of 56 600 bacterial OTUs and 7305 fungal OTUs were retained. High proportions of bacterial OTUs were classified into the phyla Proteobacteria, Actinobacteriota, and Bacteroidota (Fig. 2D), while most fungal OTUs were assigned to the phylum Ascomycota in all samples (Fig. 2J). Many differential bacterial and fungal OTUs were identified between postherbivory and normal plants (Fig. 2F and L). Further taxonomic analysis showed these OTUs were assigned to 997 bacterial genera and 288 fungal genera. The dominant genera are shown in Fig. S1. The fold change values of the top 10 genera in each microbial community were calculated to evaluate the extent of microbial composition changes (Fig. 2E and K). The relative abundance of most fungal genera (9 of 10) increased significantly in postherbivory leaves. Notably, seven of the fungal genera with increased abundance have been reported to cause several diseases in various plant species (Table S1). In particular, the genera *Alternaria* (*F* = 20.088, df = 11.763, *P* < 0.05) and *Pestalotiopsis* (*F* = 16.160, df = 9.270, *P* = 0.105) are known to cause leaf diseases in tea plants, and the relative abundance of *Alternaria sp.* was significantly increased in postherbivory leaves. It should be noted that leaf wounds after insect herbivory are conducive to the invasion, colonization, and expansion of pathogenic fungi, especially *Alternaria* species.

### Network analysis of phyllosphere endophyte microbiome from normal and postherbivory plants

Network analyses, including Molecular Ecological Networks (MENs) and Inter-Domain Ecological Networks (IDENs), were implemented to characterize interaction patterns within the endophytic phyllosphere microbial communities. Both MENs and IDENs revealed increased connectivity and complexity in the endophytic phyllosphere microbial interaction of postherbivory leaves compared to those of normal plants (Tables S2 and s3). The total nodes (bacteria networks: 574; fungal networks: 171) and links (bacterial networks: 9676; fungal networks: 833) of the microbial network within postherbivory leaves were greater than those of the normal leaves (nodes: bacterial networks: 253; fungal networks: 61; links: bacterial networks: 875; fungal networks: 73), and showed a more complex network structure. Higher node degrees (average degree: bacterial networks 33.714; fungal networks 9.743) within postherbivory leaves were observed compared to the normal leaves (average degree: bacterial networks 6.917; fungal networks 2.393), which implies that the microbial community became highly connected (Table S2). Similar results were observed in the bacterial-fungal network (Table s3), the total nodes (759) and links (6934) of the inter-domain ecological network within postherbivory leaves were greater than those of the normal leaves (nodes: 401; links: 1403), and the average degree of postherbivory and normal networks were 18.271 and 6.998, respectively.

**Figure 3:**
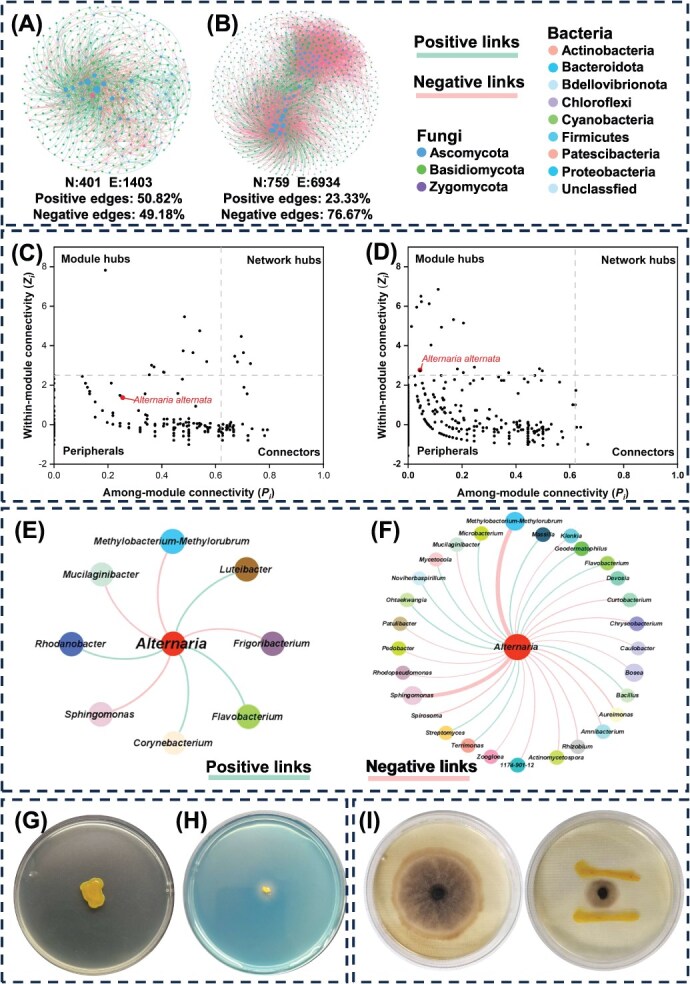
The mining and inhibition effect of crucial microorganisms and metabolites on tea leaf pathogen *A. alternata*. Inter-domain network visualization of normal samples (A) and postherbivory samples (B) (N, nodes of the network; E, edges of the network). Zi-Pi plot showing the distribution of OTUs based on their topological roles in the inter-domain ecological network of normal (C) and postherbivory (D) samples. Constructed subnetworks of *Alternaria* spp. in normal (E) and postherbivory (F) samples to mine potential antagonistic microorganisms. (G) Colony morphology of B-28. (H) Siderophore production of B-28. (I) The inhibition effects of *S. aquatilis* B-28 against the pathogenic fungus *A. alternata*.

Insect herbivory increased the positive ratio of relationships in the bacterial community from 77.83% to 99.58%, and 73.97% to 97.72% for the fungal community (Fig. S2A). However, the negative relationship ratio of inter-domain interactions between bacteria and fungi also increased dramatically, from 49.18% to 76.67% (Fig. 3A and B). Measurement of network stability indices for the MENs and IDENs indicated that the network vulnerability in postherbivory samples was lower compared to normal samples (Fig. S2B). Also, the robustness of bacterial, fungal, and bacterial-fungi community composition of postherbivory samples was significantly (*P* < 0.001) higher than in the normal group (Fig. S2B). The above results demonstrated that postherbivory increased network stability.

Zi-Pi plots were employed to explore the changes in the networks’ topology of nodes. Node topology was categorized into four types (peripherals, connectors, module hubs, network hubs) using within-module (Zi = 2.5) and among-module (Pi = 0.62) connectivity. Except for peripherals, these are considered keystone species vital to the network. One microbial species, *Alternaria alternata*, which has been repeatedly reported to cause leaf diseases in tea plants (Table S1), was identified as a keystone species in the inter-domain networks only in postherbivory samples (Fig. 3C and D), indicating this fungus had successfully invaded tea plant leaves by taking advantage of favorable conditions after insect herbivory. Considering the pathogenicity for tea plants, a strain of *A. alternata* was isolated and identified from postherbivory tea plant leaves for subsequent research (Fig. S3).

### Interactions among pathogen *A. alternata* and other microbial members

To identify endophytic phyllosphere microorganisms that may inhibit tea leaf pathogens, subnetworks of the pathogen *A. alternata* and its interacting members were constructed. Considering the strong positive correlation in the MENs networks (Fig. S2A), only IDENs were analyzed to identify potential antagonistic bacteria. The genera *Sphingomonas* and *Methylobacterium-Methylorubrum* showed negative correlations with *A. alternata*, and these negative relationships were significantly intensified in the postherbivory samples (Fig. 3E and F). Therefore, it can be supposed that these two genera may potentially have inhibitory effects on *A. alternata.* To test this speculation, the isolated strain of *A. alternata* was used as an indicator organism, and antagonistic bacteria were screened from postherbivory samples. Strain B-28, isolated and identified as *Sphingomonas aquatilis* (Fig. 3G and Fig. S4), exhibited a strong inhibitory effect, with an inhibition rate on mycelial growth of *A. alternata* of 78.65 ± 1.61% (Fig. 3I). When cultured in chrome azurol S (CAS) medium, the strain created a clear hydrolytic zone, which indicated its ability to produce siderophores (Fig. 3H), contributing to pathogen inhibition [28].

### Widely targeted-based metabolome profiling of tea plant leaves after insect herbivory

A widely targeted metabolomic analysis using UPLC–ESI–MS/MS was performed to investigate the impact of insect herbivory on the metabolite profile of tea leaves. The consistency of metabolite extraction and detection was assessed by examining the total ion current (TIC) in the quality control (QC) samples. The TIC curves of QC samples showed high overlap (Fig. S5A and B), indicating minimal technical variability in both chromatographic separation and mass spectrometric detection. Principal component analysis (PCA) was performed to assess global metabolic differences between the two sample groups. Significant differences in metabolite compositions were found, and a cumulative variance of 51.97% was explained by the first two principal components (PC1: 37.00%; PC2: 14.97%) (Fig. S5C). Furthermore, Pearson’s correlation analysis revealed significantly stronger intra-donor associations compared to inter-donor correlations across biological replicates (Fig. S5D). The experimental data revealed that the materials demonstrated consistent reproducibility, exhibiting optimal suitability for downstream analytical procedures.

A total of 1210 metabolites were tentatively identified, including 507 primary metabolites and 703 secondary metabolites. Flavonoids (318, 26.28%), phenolic acids (196, 16.20%), amino acids and derivatives (115, 9.50%), lipids (107, 8.84%), and organic acids (89, 7.36%) constituted a considerable fraction of the total metabolites (Fig. S6).

We then identified the most meaningful differentially expressed metabolites (DEMs) between control and postherbivory samples, resulting in 152 DEMs (77 upregulated, 75 downregulated) in normal and postherbivory samples (Fig. S7A). The top three upregulated metabolites in postherbivory samples were Oleanolic acid-3-O-xylosyl(1 → 3) glucuronide, 1,3-dihydroxy-4,6,8-trimethoxyxanthen-9-one and 1-α-Linolenoyl-glycerol-2,3-di-O-glucoside, while the top three downregulated metabolites were 2-Aminoethylphosphonate, pinoresinol and epipinoresinol (Fig. S7B). KEGG enrichment analysis of the DEMs provided functional insights into the metabolic changes in the different tea leaves (Fig. S8A). The DEMs were involved in 43 metabolic pathways, with the most notable being those involved in galactose metabolism and glucosinolate biosynthesis, as well as the biosynthesis of cofactors. Taken together, these results suggested that insect herbivory significantly altered the biosynthesis of metabolites in tea plant leaves.

### Transcriptomic profiling and functional annotation of tea plant leaves after insect herbivory

To probe into the molecular mechanisms of metabolic profiles in tea plant leaves damaged by insect herbivory, transcriptome analyses were performed. Data filtering produced 39.73 Gb of clean data. The transcriptomes showed consistent GC content (average 45.78%; range 45.35 to 46.27%) and high sequencing quality (Q20: 99.11 to 99.20%; Q30: 96.32 to 96.62%). A high unigene mapping rate of 93.25% to the reference genome of tea plants (https://ftp.ncbi.nlm.nih.gov/genomes/all/GCA/013/676/235/GCA_013676235.1_HZAU_G240_1.0/). further confirmed a robust assembly.

After quality assessment, a total of 732 differentially expressed genes (DEGs) were identified, with 315 upregulated and 417 downregulated (Fig. S7C). The top upregulated or downregulated genes in postherbivory samples are shown in Fig. S7D. To elucidate DEG function, we conducted a KEGG pathway analysis (Fig. S8B), which revealed significant enrichments in amino sugar and nucleotide sugar metabolism, flavonoid biosynthesis, and glucosinolate biosynthesis. Besides, GO enrichment (Fig. S9) was also performed. According to GO analysis, both the upregulated and downregulated genes in postherbivory samples were mainly concentrated in cellular anatomical entity, binding, and catalytic activity (Fig. S9A), while the top significantly enriched terms in postherbivory leaves were associated with phenylpropanoid catabolic process, lignin catabolic process, and phenylpropanoid metabolic process (Fig. S9B). Collectively, these findings indicate that gene expression in tea plant leaves during growth recovery after insect herbivory was significantly different from that in normal leaves.

### Integrated analysis of metabolome and transcriptome identifies defense and growth-promoting metabolites from tea plant leaves after insect herbivory

Differentially expressed genes and accumulated metabolites significantly affect various biological processes within plants. To evaluate the relationships between transcriptome and metabolome, the top 30 KEGG pathway enrichments for DEMs and DEGs were integrated (Fig. S8A and B). It was found that stilbenoid, diarylheptanoid, and gingerol biosynthesis, flavonoid biosynthesis, glucosinolate biosynthesis, and galactose metabolism were significantly enriched in both the metabolome and transcriptome data. These pathways are mostly associated with flavonoid and saccharide metabolism.

**Figure 4:**
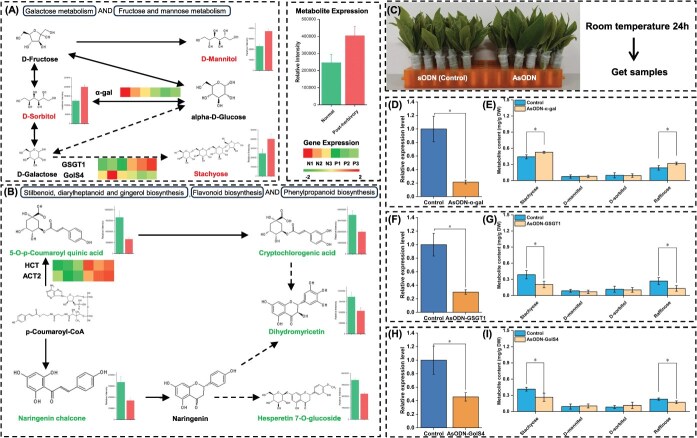
Integrated transcriptomic and metabolomic profiling and functional validation identify key metabolic pathways in tea leaves responding to insect herbivory. Integrated metabolic pathway analysis based on combined metabolomic and transcriptomic studies: (A) Galactose metabolism, fructose and mannose metabolism, and (B) Stilbenoid, diarylheptanoid, and gingerol biosynthesis, flavonoid biosynthesis, and phenylpropanoid biosynthesis. N1-3 and P1-3 stand for individual normal and postherbivory samples. (C) Schematic representation of the experimental procedure for gene silencing using AsODNs. Effects of AsODN-mediated gene suppression and carbohydrate metabolism compared to scrambled control (sODN) of *α-gal* (D and E), *GSGT* (F and G), and *GolS4* (H and I) genes. The bar chart represents the expression levels of the corresponding metabolites, with error bars representing the standard deviation (±SD). The symbol * indicates a statistically significant difference between two groups (*P* < 0.05).

D-sorbitol and stachyose were significantly upregulated in the metabolic pathways of galactose metabolism, and D-mannitol was also upregulated in a closely linked metabolic pathway of fructose and mannose metabolism (Fig. 4A). Also, a probable GSGT1 gene (probable galactinol-sucrose galactosyltransferase1) and GolS4-like gene (galactinol synthase 4-like) may play important regulatory roles in stachyose synthesis, while α-gal-like gene (alpha-galactosidase-like) may play a key role in the transformation between D-sorbitol and D-mannitol (Fig. 4A). As these three metabolites are known to be important for plant growth [29, 30], it can be supposed that these metabolites facilitate the postherbivory recovery of tea plant leaves.

In addition, five metabolites (5-O-p-Coumaroylquinic acid, cryptochlorogenic acid, dihydromyricetin, naringenin chalcone and hesperetin 7-O-glucoside) were observed to decrease significantly in the pathways of stilbenoid, diarylheptanoid, and gingerol biosynthesis, flavonoid biosynthesis, and phenylpropanoid biosynthesis (Fig. 4B). The HCT-like gene (shikimate O-hydroxycinnamoyltransferase-like) and ACT-like gene may play important roles in downregulating these metabolites (Fig. 4B). The critical roles of flavonoids and phenolic acids in antifungal activity and plant disease resistance mechanisms have been well-documented in recent studies [31, 32]. It is supposed that these compounds may demonstrate potential inhibitory effects against tea leaf pathogens.

### Effects of silencing *GSGT1*, *GolS4*, and *α-gal* on stachyose, mannitol, and sorbitol accumulation

To further characterize the functions of *α-gal*, *GSGT1*, and *GolS4* in carbohydrate metabolism, antisense oligonucleotides (AsODNs) were synthesized to inhibit their expression in tea plants (Fig. 4C). The expression levels of these genes following AsODN treatment was quantified to evaluate silencing efficiency (Fig. 4D, F, and  H). Quantitative RT-PCR confirmed that the AsODN treatment reduced the expression of *α-gal*, *GSGT1*, and *GolS4* to 21.5%, 29.9% and 45.7% of the control (*P* < 0.05), respectively. Silencing of *α-gal* obviously increased the contents of stachyose and raffinose (Fig. 4E), while silencing of *GolS4* and *GSGT1* significantly decreased the accumulation of stachyose and raffinose (Fig. 4G and I). However, these three genes exhibited no significant regulatory effects on other related metabolite biosynthesis, such as mannitol and sorbitol (Fig. 4E, G, and  I).

### Functional verification test of mined metabolites

**Figure 5:**
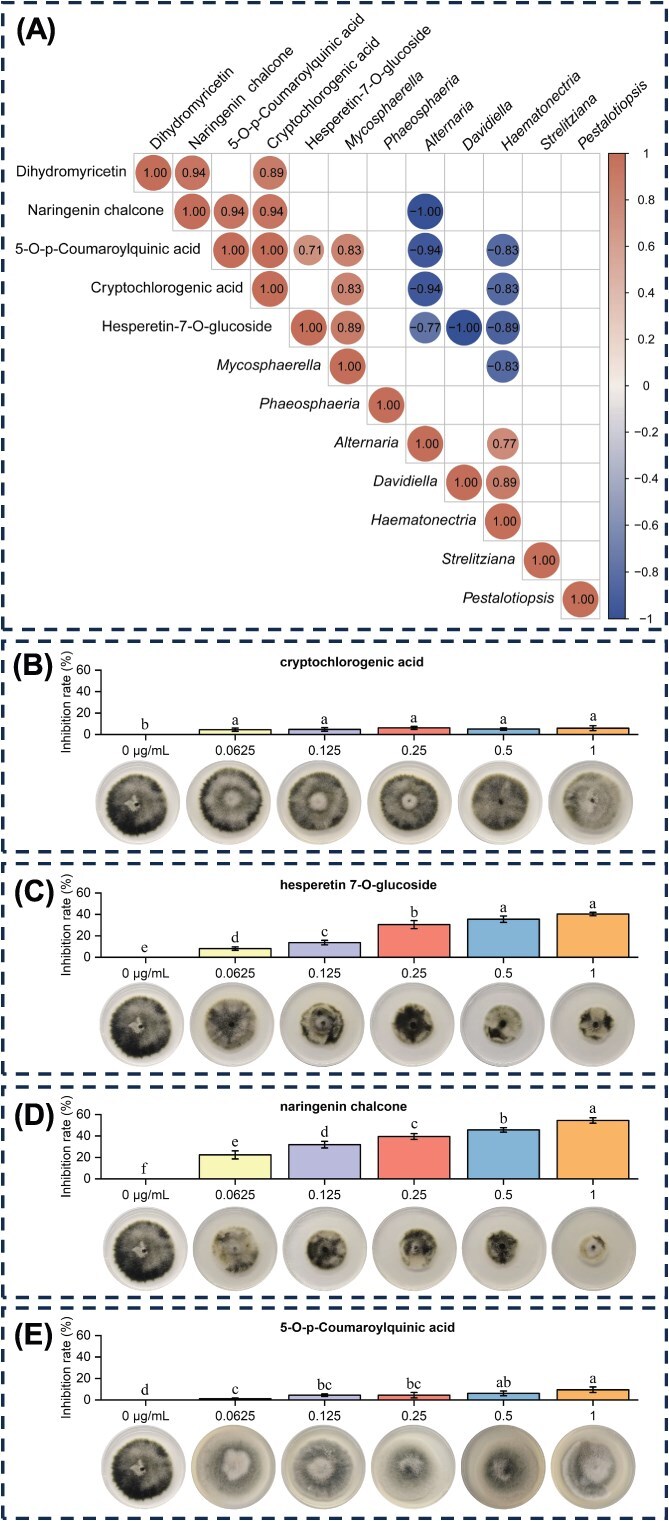
The mining of defense metabolites based on correlation analysis and their inhibition effect on tea leaf pathogen *A. alternata*. (A) Integrated analysis of microbiome and metabolome based on Spearman correlation analysis between potential defense metabolites and seven genera (top ten) of plant pathogens. The inhibition effects of cryptochlorogenic acid (B), hesperetin 7-O-glucoside (C), naringenin chalcone (D), and 5-O-p-Coumaroylquinic acid (E) on pathogen *A. alternata*. Error bars represent standard deviation (± SD). All results were obtained from three independent experiments, with five replicate plates included per experiment. Different lowercase letters represent significant differences between different groups (*P* < 0.05).

Integrated analysis of microbiome and metabolome based on Spearman correlation analysis between potential defense metabolites and *A. alternata* indicated that hesperetin 7-O-glucoside, naringenin chalcone, cryptochlorogenic acid, and 5-O-p-Coumaroylquinic acid may inhibit the growth of *A. alternata* (Fig. 5A). To further confirm the potential functions of the mined metabolites in resisting the invasion of pathogens in tea plant leaves after insect herbivory, plate inhibition tests and field growth promotion experiments with different concentrations of metabolites were conducted. Plate inhibition testing (Fig. 5C and D) showed that hesperetin 7-O-glucoside and naringenin chalcone have good inhibitory effects on the pathogen *A. alternata* at various concentrations, and their highest inhibitory rates were 40.34% and 54.52% at the concentration of 1.0 μg/ml, respectively. Cryptochlorogenic acid and 5-O-p-Coumaroylquinic acid showed only a minor inhibition effect on *A. alternata* (Fig. 5B and E).

**Figure 6:**
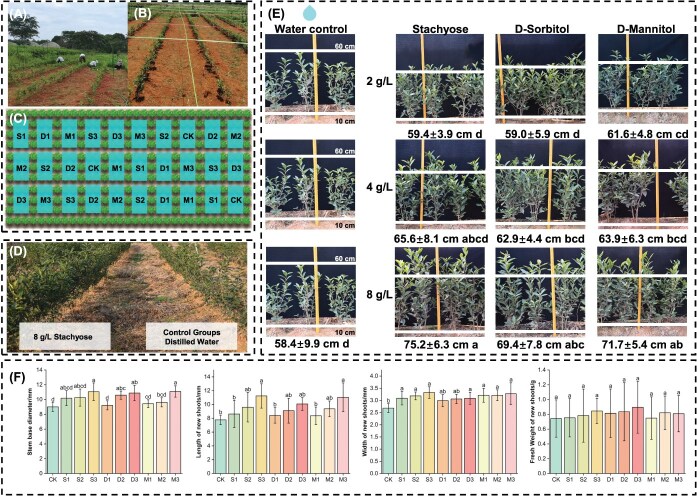
Field growth-promoting effect of key metabolites on tea plants. (A–C) Experimental layout and design. S1, S2, and S3 represent stachyose concentrations of 2 g/L, 4 g/L, and 8 g/L, respectively. D (D-sorbitol) and M (D-mannitol) are the same as stachyose. (D) Comparison of actual growth-promoting effect. (E) Effects of different metabolite concentrations on the plant height of tea plants (Mean ± SD). (F) Effects of different metabolite concentrations on tea plant biomass, with error bars representing ± SD. Measurements were performed on 30 individual plants within a single plot. Different lowercase letters represent significant differences between different groups (*P* < 0.05).

Integrated analysis of transcriptome and metabolome indicated that D-sorbitol, stachyose and D-mannitol have the potential to promote growth recovery in tea plant leaves after insect herbivory. Further field-promoting experiments showed that low concentrations of D-sorbitol, stachyose and D-mannitol have no significant effect on the agronomic traits of tea plants, while medium and high concentrations of these metabolites could significantly improve the agronomic traits of tea plants (Fig. 6E and F). The 8 g/L stachyose treatment showed the most significant growth-promoting effect, with an increase of 22.34%, 22.71%, 13.77%, 44.66%, and 27.76% in tea plant height (*P* < 0.05), stem base diameter (*P* < 0.05), fresh weight of new shoots, length of new shoots (*P* < 0.05), and width of new shoots (*P* < 0.05), respectively.

## Discussion

### Insect herbivory induced endogenous pathogenic fungi to occupy the favorable ecological niche

Insect herbivory triggers multilevel restructuring of plant-associated microbial communities, with perturbations extending beyond the rhizosphere and phyllosphere to profoundly reshape endophytic microbiota residing within plant tissues [24, 33]. As symbiotic microorganisms, endophytes asymptomatically colonize plant tissues during critical phases of their life cycle [22]. Commonly, fungal endophytes exhibit a functional spectrum spanning from latent pathogens to constitutive mutualists, achieving a steady state by residing in equilibrium with their host’s tissues [34]. However, these potential pathogens may also be transformed into host opportunistic pathogens when under certain conditions, especially under biotic and abiotic stress [35, 36]. These endophytic microorganisms remain at low abundances in healthy plants due to the dual constraints of low nutrient availability in the apoplast and constitutive immune suppression [37]. However, following insect postherbivory, substantial amounts of nutrients are released into the apoplast [38]. Concurrently, plants reallocate their metabolic resources from defense to growth recovery, which is accompanied by a coordinated downregulation of defense-related metabolites [39]. Consequently, the alleviation of both nutrient limitation and plant defense pressure leads to a significant increase in pathogen abundance. As observed in our research, the composition of postherbivory tea plant leaves had an enhanced relative abundance of pathogens (Fig. 2K), especially *Alternaria* sp. (*P* < 0.05), which are reported to cause leaf disease of tea plants (Table S1). In addition, according to the topological role of *A. alternata* in inter-domain networks, this pathogen changed from a peripheral in normal leaves to module hub in postherbivory leaves (Fig. 3C and D), where it behaved as a keystone species. Core microbiomes establish the foundational niches that facilitate their colonization [40]. The observed topological role changes in the node of *A. alternata* suggest a potential ecological risk that endogenous pathogenic fungi may preferentially colonize insect stress-induced advantageous niches [41], which potentially increases the risk of leaf disease outbreak. Notably, *B. melanopus*, a pest that causes mechanical damage to tea leaves, emerges from the soil and initiates feeding when ambient temperatures are sustained above 20°C. The optimal growth range of *A. alternata* is 20–25°C [42], a thermal range fully overlapping with the peak activity of *B. melanopus* in tea plantations. Such tight temperature synchrony, alongside herbivory-induced nutrient release, generates pronounced synergism. This creates ideal conditions for the proliferation and pathogenesis of latent *A. alternata*. Driven by combined biotic stress, suitable abiotic environments and adequate nutrients, the pathogen shifts from a minor endophyte to a dominant niche occupant. Accordingly, insect herbivory substantially raises the risk of endogenous fungal disease outbreaks in tea plantations.

Restructuring endophytic microbiota compensates for the defense of tea plants during postherbivory recovery phases

Phyllospheric microbial communities play a crucial role in maintaining plant health [43]. The maintenance of physiological stability of phyllospheric microbiota not only inhibits pathogens and enhances plant biotic resistance, but also boosts photosynthesis and secondary metabolism, thereby improving productivity [43–45]. Microbial diversity serves as the core driver of ecosystem multifunctionality [46]; its species richness enhances resource utilization efficiency through ecological niche differentiation [47], while functional redundancy strengthens system stability [48]. These two mechanisms synergistically support multidimensional ecological processes, especially plant disease resistance regulation [49]. Thus, microbial diversity of the plant rhizosphere and phyllosphere has been recognized as a key reference index in resisting plant diseases. Evidence from prior studies [46–49] supports the idea that postherbivory leaves’ high microbial diversity can be crucial in effectively suppressing the pathogen *A. alternata* infestation in our results (Fig. 2A, B, G, and  H).

Co-occurrence networks are widely applied to identify key antagonistic microorganisms in complex interaction relationships [26, 50]. In postherbivory samples, *Sphingomonas sp.* exhibited a more enhanced negative correlation with the pathogen *A. alternata* compared to normal samples (Fig. 3E and F). Furthermore, the isolated beneficial strain B-28 (*S. aquatilis*) was demonstrated to significantly inhibit the mycelial growth of *A. alternata* (Fig. 3I). Moreover, it was also found that D-mannitol, which underwent upregulated biosynthesis, was a better carbon source for *S. aquatilis* growth than glucose at 72 h postinoculation (Fig. S10). Moreover, absolute quantification revealed that the abundance of *Sphingomonas sp*. was significantly higher in postherbivory samples (Fig. S11). This means that tea plant metabolic resources reallocated to growth can indirectly enhance defense by favoring the growth of antagonistic microbes, thereby reducing defense costs while prioritizing growth. In addition, microbial network structure and topological information can be used to evaluate the stability of the microbial community [51]. Our results indicated that the microbial community of postherbivory leaves exhibits stronger robustness and lower vulnerability, which represents a more stable network and is generally considered more disease-resistant [52, 53] (Fig. S2B). Previous studies have shown the crucial role of plant-associated microbes in decoupling the growth-defense trade-offs through growth promotion or pathogen inhibition [54], which is in line with our results that the restructured endophytic microbiota compensates for the defense of tea plants during postherbivory recovery phases, ultimately inhibiting leaf disease outbreaks.

### Tea plants prioritize growth over defense through metabolic resetting during postherbivory recovery phases

Plants are exposed to insect herbivory and pathogen invasion throughout their life cycle, and must face various and changeable survival threats [55]. In response to continuous biotic threats, plants have developed highly sophisticated defense mechanisms that ensure their effective protection [56]. But these defense mechanisms require an enhanced energy supply, usually at the cost of reduced plant growth [11]. Therefore, plants have developed a growth-defense trade-off to balance plant growth and disease resistance [57], minimize defense costs, and stimulate their own growth when in the recovery period after environmental stress [58]. Our results showed that tea plants downregulated defense metabolites and initiated a saccharide-led recovery strategy to prioritize growth. The inducible defense metabolites of plants, such as flavonoids, phenolic contents, jasmonic acid, and salicylic acid, could defend against pathogen invasion, but also have adverse effects on plants, such as toxicity and excessive energy consumption [59, 60]. Compared to normal samples, these potential defensive compounds were down to a low level or returned to reasonable levels, especially the flavonoids (24 out of 33 flavonoid metabolites downregulated, Table S4), which was attributed to either excessive consumption during the early defense stage or a growth-defense trade-off strategy that prioritizes plant growth. In addition, through systematic joint analysis of multiomics datasets, four critical defense compounds were identified, verified, and evaluated, with naringenin chalcone and hesperetin-7-O-glucoside demonstrating potent inhibitory activity against the pathogen *A. alternata* (Fig. 5C and D).

Moreover, saccharides were well recognized as playing a crucial role in the development, growth, and stress response of plants [61]. Our results indicated that upregulated metabolic saccharides (all six saccharide metabolites were upregulated in the current study, Table S5) may be responsible for driving tea plant growth recovery. The field experiments also verified that three saccharide substances (stachyose, D-sorbitol, and D-mannitol) can significantly promote the biomass increase of tea plants at appropriate concentrations (Fig. 6D, E, and  F). It is well known that plants face trade-offs between growth and defense, with their potential for defense being negatively correlated with growth [58]. Our results demonstrated the downregulation of defensive metabolites to facilitate the production of growth-promoting metabolites during the plant recovery stage, which is highly consistent with the plant growth-defense trade-off mechanism. This intricate mechanism enables plants to respond rapidly and efficiently to adverse stresses, thereby ensuring their survival [62]. However, this impedes the development of ultrahigh-yielding crops, as both enhanced productivity and stress resilience are required simultaneously [63].

Saccharides, as products of photosynthesis, play crucial roles throughout the entire life cycle of plants, providing energy and carbon skeletons for plant growth and development [61, 64]. Saccharides promote plant growth through various mechanisms [65], and, in this study, three types of saccharides (D-sorbitol, stachyose and D-mannitol) displayed unique mechanisms in promoting plant growth. D-mannitol and D-sorbitol can serve as saccharide reserves for energy storage and compound transfer, as well as for enhancing plant growth [66, 67]. Stachyose functions as a signaling molecule during pathogen attack and insect-led wounding, is involved in saccharide catabolism to generate energy, and plays key roles in plant growth and development [68]. Overall, it was found that tea plants prioritize growth over defense through metabolic resetting.

### Gene expression modulates saccharide-related metabolic pathways of postherbivory tea plant leaves

Through transcriptomics analysis of normal and postherbivory leaves, multiple differentially expressed genes were identified. The probable galactinol-sucrose galactosyltransferase1 (probable GSGT1, LOC114314766), galactinol synthase 4-like (GolS4-like, LOC114282083), and alpha-galactosidase-like (α-gal-like, LOC114290167) genes may play critical regulatory roles in stachyose synthesis, as evidenced by the integrated transcriptomic and metabolomic analysis. During stachyose metabolism, the expression of GolS4-like and α-gal-like genes was repressed in postherbivory samples, while the expression of the probable GSGT1 gene was promoted. The GolS gene initiates the biosynthesis of raffinose family oligosaccharides [69], and the α-galactosidases regulated by the α-gal gene catalyze the first catabolic step of RFOs [70]. The downregulation of these two genes suggests an impairment in both the synthesis and degradation of RFOs. By silencing the *α-gal* gene, we confirmed its role in RFOs degradation, as evidenced by stachyose and raffinose increase after the *α-gal* gene was silenced (Fig. 4E). Silencing the *GolS* gene inhibited stachyose biosynthesis, further confirming the role of the *GolS* gene in RFO synthesis (Fig. 4I). In addition, after silencing the *GolS* gene, we observed an increase in the content of raffinose (Fig. 4I). This observation differs from previous studies and highlights the complexity of the carbohydrate regulatory network [71, 72]. Moreover, the upregulated GSGT1 is a key enzyme in the biosynthesis of RFOs, which synthesizes raffinose by transferring a galactose unit from 1-α-D-galactosyl-myo-inositol to sucrose [73], and raffinose could rapidly be converted to stachyose [74]. The critical role of *GSGT1* in stachyose synthesis was also confirmed. Silencing the *GSGT1* gene led to a significant reduction in stachyose and raffinose content (Fig. 4D). Furthermore, the decrease in expression of α-gal-like genes responsible for catabolism of RFOs leads to a persistent stachyose accumulation, thereby creating a feedback loop that downregulates the expression of the GolS4-like gene and the RFOs biosynthesis. Metabolomic analysis revealed a significant accumulation of stachyose in postherbivory leaves, which is consistent with transcriptomic results. As a member of the raffinose family oligosaccharides, the accumulation of stachyose in the plant recovery stage after insect herbivory plays a key role in plant growth and development [68], and was supported by our observation of field experiment results (Fig. 6). While we have explored and discussed the potential functions of these differentially expressed genes in saccharide regulation, the molecular mechanisms in regulating the growth-defense trade-offs remain to be fully elucidated, as this endeavor may be constrained by the molecular assays employed in this study and the precision of metabolite detection. Future studies should focus on elucidating the signaling pathways and regulatory networks involving these genes to better understand how plants promote growth in the recovery stage.

**Figure 7:**
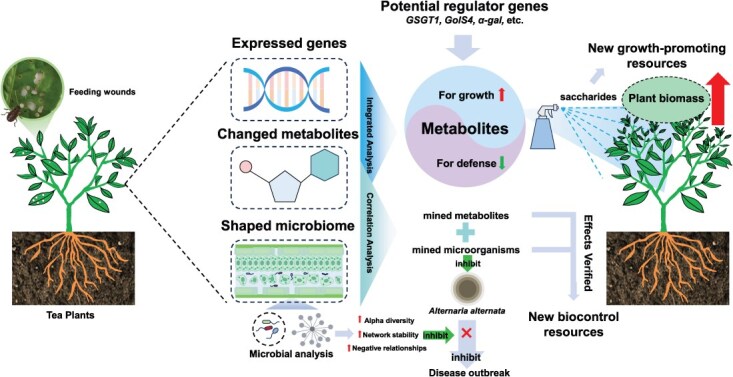
The growth and defense trade-off mechanism of tea plant leaves during the recovery growth stage after insect herbivory.

In summary, the underlying mechanism of growth-defense resource reallocation in *C. sinensis* during postherbivory ecological compensation processes was elucidated through multiomics analyses integrating the microbiome, metabolome, and transcriptome. During the recovery stage after insect herbivory, a significant enrichment of the tea leaf pathogen *A. alternata* was observed, which may be driven by the downregulation of defense metabolites. However, the microbial community in postherbivory samples exhibited a higher α-diversity and a more complex interaction network. Interestingly, the beneficial microbes revealed through network analysis demonstrated strong inhibitory effects on *A. alternata.* Restructuring of the endophytic microbiota potentially inhibits leaf disease outbreaks. An integrated metabolomic and transcriptomic approach highlighted that genes like *GSGT1*, *GolS4*, and *α-gal* might regulate synthesis and degradation of growth-promoting and defense metabolites. The downregulated flavonoids were defense compounds against pathogens and upregulated saccharides were plant growth-promoting compounds, which were verified in subsequent tests. Overall, tea plants compensate for the reduction in defense through restructuring of the endophytic microbiota and by prioritizing growth over defense through metabolic resetting. However, this study only sampled a single time point after insect herbivory, limiting our observation of the transition from defense to growth. Further time-series experiments are therefore needed to track the shifts between growth and defense following insect herbivory. Simultaneously, this study also uncovered new biocontrol and growth-promoting resources, providing support for understanding the compensation strategies of tea plants after insect herbivory (Fig. 7).

## Materials and methods

### Experiment design

A controlled field experiment was conducted in a tea plantation in Chenzhou (112°55′56″E, 25°47′6”N), Hunan province, China, where *B. melanopus* herbivory was prevalent. This pest overwinters as larvae within the root-zone soils of tea plants and becomes active from mid-April to early June, during which the adults damage tea plant leaves. The tea plant variety Zhuyeqi with an age of more than 5 years was used for this experiment. To prevent adults from emerging from pupae within the soil, plastic mulch was applied to the soil surface before insect outbreaks. Additionally, insect-proof nets were installed to prevent the migration of pests from external sources. The experiment consisted of two types of plots, each covering an area of 30 m^2^. One was regarded as the control plot, and the other was regarded as the postherbivory plot, with three replicate plots created for each type. During the peak period of adult emergence, *B. melanopus* were collected from the tea plantation and released into the postherbivory plot at a density of 2000 individuals based on field observations of natural population levels. The insects were allowed to feed freely on the tea leaves for three days, after which all introduced insects were manually recaptured and eliminated. Once no further pest damage was observed, the plastic mulch and insect-proof nets were removed, exposing the tea plants to natural environmental conditions. The experimental design and its intuitive presentation are shown in Fig. 1.

### Sampling

Tea leaves from each plot were subsampled for the assessment of multiple response variables, including high-throughput sequencing, metabolome and transcriptome analysis. On July 1, 2022 (six weeks after insect herbivory, 42 days), 8–12 leaves were carefully collected from nearly identical positions of more than three plants within each replicate plot of each type to create a single composite sample, and twenty samples (10 from the postherbivory plots and 10 from the control plots) were collected. Following surface sterilization in 75% ethanol (90 s), 3.25% sodium hypochlorite (3 min), and a second 75% ethanol wash (30 s), the damaged leaves (25–30 holes each) were rinsed thrice in sterile water, dried on filter paper, flash-frozen in liquid nitrogen, and stored at −80°C for subsequent analysis.

### High-throughput sequencing analyses of leaf endophytic microbiome

Twenty complex samples were used for high-throughput analyses, ten from the postherbivory plot and ten from the normal plot. After extraction with the FastDNA Spin Kit (MP Biomedicals), the total DNA from leaf samples was assessed for concentration and quality using a Nanodrop One spectrophotometer (Thermo Scientific). For taxonomic profiling, universal primers were employed to amplify specific gene regions. These included the V5-V6 hypervariable region of the bacterial 16S rRNA genes (799F: 5′-AACMGGATTAGATACCCKG-3′, 1115R: 5′-AGGGTTGCGCTCGTTG-3′), and the Internal Transcribed Spacer 1–2 region genes of fungi (ITS1: 5′-CTTGGTCATTTAGAGGAAGTAA-3′, ITS2: 5′-GCTGCGTTCTTCATCGATGC-3′). Sample-specific barcodes were added to these primers to distinguish different samples. Finally, the samples were sequenced on the Illumina Nova 6000 platform.

All raw sequence data were processed with a publicly accessible pipeline (https://dmap.denglab.org.cn/) that integrates diverse bioinformatics tools [75]. Firstly, all raw reads were uploaded, and barcodes were employed to assign them to samples, permitting one mismatch. FLASH was used to combine forward with reverse sequences. The UPARSE algorithm was used to remove chimeras and generate an operational taxonomic units (OTUs) table at a 97% threshold [76]. The taxonomic information of bacterial and fungal representative sequences was assigned by the Ribosomal Database Project (RDP) classifier based on the SILVA database and UNITE database, respectively [77, 78]. The sequences were further filtered to remove nonbacterial or nonfungal sequences.

MENs and IDENs were constructed via a random matrix theory (RMT)-based approach to elucidate the relationships between the genera *Alternaria* and endophytic leaf microbial communities. Network analysis was carried out using a publicly available pipeline https://inap.denglab.org.cn/ [79]. Using the Maslov-Sneppen procedure [80], we generated 100 random networks for each observed network and evaluated their topological properties to assess complexity and vulnerability. Robustness and vulnerability were used to assess the stability of MENs and IDENs impacted by insect herbivory. Robustness was calculated by determining the proportion of species that remain in the network following the removal of random or targeted nodes [81]. Vulnerability assesses the vulnerability of each node to quantify its relative contribution to the overall network efficiency [81]. The constructed networks of endophytic leaf communities in different samples were visualized by Gephi 0.9.2 and Cytoscape 3.8.2.

### Targeted strains isolation from the endophytic phyllosphere

Through in-depth mining of microbiome data, the enrichment of pathogens in tea plant leaves and their negative relationships with antagonistic microbes were discovered in postherbivory leaves. To obtain target endophytic pathogenic fungal strains, fresh tea leaf samples were isolated after surface sterilization [82]. To obtain target fungi, the sterilized tea leaf segments (5 mm × 5 mm) were seeded onto the surface of potato dextrose agar (PDA), Martin agar, Czapek-Dox agar and Malt Extract Agar (MEA) plates supplemented with 0.05 g streptomycin sulfate per 100 ml to inhibit bacterial growth. For target beneficial bacteria, Lysogeny Broth (LB), Tryptic Soy Agar (TSA), R2A Agar and Nutrient Agar (NA) media were prepared for isolation. The plates were incubated at 28°C and checked daily for any fungal or bacterial growth. Individual isolates grown out from the tissues were picked out using the tip of an inoculation needle and transferred to the center of another fresh PDA or LB medium plate for purification. Purified strains were placed on a slant at 4°C and stored until further testing.

### Isolate identification and phylogenetic analysis

The genomic DNA of the purified strains was extracted by the cetyltrimethylammonium bromide (CTAB) method. The primers ITS1/ITS4 (ITS1: 5′-TCC GTA GGT GAA CCT GCG G-3′; ITS4: 5′-TCC TCC GCT TAT TGA TAT GC-3′) and GPD1/GPD2 (GPD1: 5′-CAA CGG CTT CGG TCG CAT TG-3′; GPD2: GCC AAG CAG TTG GTT GTG C-3′) were used to amplify the internal transcribed spacer (ITS) and glyceraldehyde 3-phosphate dehydrogenase (GAPDH) for pathogenic fungi in a thermocycler, while the 16S rDNA gene was amplified using the primers 27F/1492R (27F: 5′-AGAGTTTGATCCTGGCTCAG-3′; 1492R: 5′-CTACGGCTACCTTGTTACGA-3′) to identify potential antagonistic bacteria.

The purified strain sequences were subjected to BLAST analysis to confirm the genera, and the sequences of the corresponding type strains were gathered for each genus (Table S6). Subsequently, neighbor-joining phylogenetic analysis was carried out for the sequences using MEGA 7 to determine the species information of purified strains.

### Antagonistic activities of bacteria against *A. alternata*

To assess antagonism, bacterial isolates were streaked on two sides of LB plates, with a central plug of in situ-isolated *A. alternata* mycelium (d = 5 mm) placed between them. All plates (five replicates), including *Alternaria* only controls, were incubated at 25°C for 7 days, after which the percent inhibition of fungal mycelial growth was quantified as follows: 100 × [(d1 – d2)/d1], where d1 was the radial growth of *A. alternata* in control cultures, and d2 was radial growth of *A. alternata* in *A. alternata*–antagonistic bacteria dual cultures [83]. All experiments were performed three times independently.

### Quantification of the absolute abundance of *Sphingomonas sp.*

Absolute abundance of *Sphingomonas* was determined by real-time quantitative PCR according to a previous study [84]. The 16S rRNA gene of *S. aquatilis* was amplified with the genus-specific primers SA429f/SA933r (SA429f: 5′-TAAAGCTCTTTTACCCG-3′; SA933r: 5′-TAAAGCTCTTTTACCCG-3′). The PCR products were cloned into the pUC19 vector and transformed into *E. coli* DH5α. Recombinant plasmids were linearized with *Eco*R I, and purified DNA concentrations were quantified using an Eppendorf spectrophotometer. The plasmid copy number was calculated using the following formula: plasmid copy number (copies/μl) = [6.02 × 10^23^ × plasmid concentration (ng/μl) × 10^−9^]/(plasmid length × 660). A series of 10-fold serial dilutions of linearized plasmids were assayed in triplicate by real-time PCR to establish the external standard curve.

### Metabolite measurement and metabolome analysis

Three corresponding postherbivory and normal samples above were also conducted for additional metabolome and transcriptome analysis. The widely targeted metabolomics method, a recently developed efficient technique for the identification and quantitation of metabolites that has been applied to explore the interactions between tea plants and pests research [85], was employed to compare changes in metabolites in postherbivory and normal samples. All sample analyses were performed as described previously [86, 87]. In brief, after lyophilization (Scientz-100F) and grinding (MM 400 mixer mill; 30 Hz, 1.5 min), 50 mg of processed leaf powder was methanol-extracted at 4°C with periodic vortexing. The centrifuged supernatant (10 000 × g, 10 min) was filtered (0.22 μm) prior to UPLC–MS/MS analysis. Metabolite quantification employed scheduled multiple reaction monitoring. Metabolites were identified by analyzing the tandem mass spectra of ions detected by MS. The fold change value for each metabolite was calculated as the ratio of the scaled amount of postherbivory samples relative to that of the normal samples. Differential metabolites were identified by combining a significant VIP threshold from the OPLS-DA model with *P* values from a two-tailed Student’s *t*-test, and the FC (fold change) value was also taken into consideration as one of the crucial indicators. Thus, metabolites with VIP > 1.0, *P* < 0.05 and absolute Log_2_FC > 1.5 were considered as DEMs.

### Transcriptome profiling

Transcriptome profiling was performed as described previously [88]. In brief, total RNA was extracted from the independent postherbivory and normal samples (three biological replicates were performed for transcriptome profiling). Raw reads from the Illumina HiSeq sequencing platform were processed with fastp v0.23.2 to trim adapters and remove low-quality and short sequences. Filtered reads were aligned to the *C. sinensis* reference genome (GCA_013676235.1) with Hisat2 v2.2.1 under default parameters. RSEM v1.3.3 was employed to estimate the fragments per kilobase of transcript per million fragments mapped (FPKM), and differentially expressed genes (DEGs) were identified by DESeq2 v1.34.0 with a threshold of absolute Log_2_FC (fold change) >1.5, *P* < 0.05 and FDR < 0.05.

### The exploration of potential functions of differential metabolism

A Spearman rank correlation analysis was conducted between potential defense metabolites and seven plant pathogen genera (in the top ten) to explore the potential antifungal metabolites. Screened metabolites, which show negative correlation with the relative abundance of *A. alternata*, including 5-O-p-coumaroylquinic acid, cryptochlorogenic acid, naringenin chalcone and hesperetin-7-O-glucoside, were selected for pathogen inhibition experiments. All the analytical reagents were above the chromatographic purity level. For the antifungal assay, different concentrations of the above compounds were prepared (0 μg/ml, 0.0625 μg/ml, 0.125 μg/ml, 0.25 μg/ml, 0.5 μg/ml and 1.0 μg/ml) in a volume of anhydrous ethanol. Pathogen inhibition activity was tested using PDA agar plates containing the drugs, and the mycelial growth inhibition effects were calculated as described previously [83].

Significantly upregulated metabolites, including D-mannitol (Sigma, 98%), D-sorbitol (Macklin, 98%) and stachyose (Aladdin, 98%), were selected for tea plant growth promotion field experiments. To verify their actual plant growth-promoting effects, field experiments were further conducted in a tea plantation on July 1, 2024, in Changsha (113°14′2″E, 28°09′13”N), Hunan province, China. The experiment site consisted of thirty 3 × 3 m^2^ plots. Different metabolites (D-Mannitol, D-Sorbitol and Stachyose) of different concentrations (2 g/L, 4 g/L, 8 g/L) were foliar sprayed in different experimental plots, and distilled water without any metabolites served as the control. Three replicates were used for each treatment, and at least 30 tea trees were planted in each plot. The first foliar spraying and soil irrigation were performed on July 1, 2024, with the metabolites dissolved in distilled water. Subsequently, two additional sprayings and irrigations were performed on July 15 and August 1, 2024. The tea plants’ biomass was measured on December 20, 2024.

### The growth-promoting effects of saccharides on *S. aquatilis*

Minimal medium was used to assess the growth-promoting effects of D-mannitol, D-sorbitol and stachyose on *S. aquatilis*. To find the optimal concentration responsible for the growth of *S. aquatilis*, various concentrations (5, 6, 7, 8, 9, and 10 g/L) for each saccharide were individually supplemented to the minimal medium, replacing glucose. Additionally, these three saccharides were added to 200 ml minimal liquid medium at the optimal concentration above to evaluate the utilization efficiency of different carbon sources. After being cultured at 28°C with a shaker speed of 180 rpm for 12, 24, 48, and 72 h, the OD_600_ values were measured using a spectrophotometer, with each group having five replicates. All experiments were performed three times independently.

### Silencing of genes in tea plant leaves

AsODNs were designed using Soligo software to inhibit the expression of *GSGT1*, *GolS4*, and *α-gal* [89]. Tender shoots of tea plants were treated with gene-specific 20 μM AsODNs, while a separate group was treated with a sense strand solution (sODN) as a control. After 24 h of silencing treatments, samples were collected for gene expression analysis and key metabolic changes.

Total RNA was extracted from tea leaves using the FastPure Universal Plant Total RNA Isolation Kit. The RNA was reverse transcribed into cDNA using the FastKing One- step RT-PCR Kit, with 1000 ng of total RNA as the template. The resulting cDNA was used as a template for RT-PCR analysis, which was performed using the SYBR Green Premix Pro Taq HS qPCR kit (Agbio, China) on a QuantStudio 1 Plus system (Thermo Fisher Scientific, USA). Gene expression levels in tea plants were normalized to the *β-actin* gene using the 2^-ΔΔCT^ method, with three biological replicates for the experiment. The primers used are listed in Table S7.

Target metabolites (D-mannitol, D-sorbitol, stachyose) and the relevant carbohydrate were quantified by HPLC-ELSD (Shimadzu LC-20AT, Japan; AllChrom ELSD 6000, China). Samples were separated on an Rocksil Carbohydrate ES column (250 mm × 4.6 mm). Isocratic elution was utilized with ultrapure water and acetonitrile (24:76, v/v) as eluents, with a flow rate of 1.0 ml/min.

### Statistical analyses

The observed significant differences in the statistical analysis were evaluated using a two-tailed Student’s *t*-test (for two groups) or the ANOVA test (for more than two groups).

## Supplementary Material

Web_Material_uhag338

## Data Availability

All raw data of the microbiome were submitted to the National Center for Biotechnology Information (NCBI; https://www.ncbi.nlm.nih.gov/) under the BioProject ID: PRJNA1281994 (16S, SRA accession numbers for each sample: SRX29352468 to SRX29352487) and PRJNA1282012 (ITS, SRA accession numbers for each sample: SRX29353918 to SRX29353937). All raw data of the transcriptome were submitted to the National Center for Biotechnology Information (NCBI; https://www.ncbi.nlm.nih.gov/) under the BioProject ID: PRJNA1283354 (SRA accession numbers for each sample: SRX29453601 to SRX29453606). All raw data of the metabolome were submitted to the MetaboLights (https://www.ebi.ac.uk/metabolights/) under accession number MTBLS12664. The obtained sequences of isolates were submitted to the National Center for Biotechnology Information (NCBI; https://www.ncbi.nlm.nih.gov/) GenBank for accession numbers (*Alternaria alternata* CY-6: ITS: PV450547; GAPDH: PV468350; S*phingomonas aquatilis* B-28: 16S: PV451605).
