# Supplementary material for: Multiomics reveals postherbivory tea leaves restructure endophytic microbiota to resist disease and boost saccharide metabolism to promote growth
Source: Hortic Res. 2026 Aug 4;13(8):uhag338. doi: 10.1093/hr/uhag338 (PMC13482628; doi:10.1093/hr/uhag338)
Supplement: Supplementary file 1 [file web_material_uhag338.zip › Supplementary Figure.docx]

**Supplementary Figures**

**Figure S1. Relative abundance of the top ten phyla and genera.** **(A)** and **(B)** represent the top ten phyla and genera of phyllosphere bacteria, respectively. **(C)** and **(D)** represent the top ten phyla and genera of phyllosphere fungi, respectively.

**Figure S2. Structural and functional dynamics of microbial networks in response to insect herbivory.** **(A)** Network visualization of normal samples and post-herbivory samples (N, nodes of the network; E, edges of the network). **(B)** Network stability index of molecular ecological networks and inter-domain ecological networks.

**Figure S3. Identification of *Alternaria alternata* by morphology and molecular biology. (A)** The neighbor-joining phylogenetic tree constructed with the combination of ITS (579bp) and GAPDH (501bp) sequences of type strains of species within *Alternaria*. The phylogenetic tree was rooted with the outgroup *Bipolaris cynodontis* CBS 109894. GenBank accession numbers are provided in Table S5. T indicates type strain. The conidia **(B)** and perithecium **(C)** of the *A. alternata* isolate CY-6 from this study.

**Figure S4. Identification of potential antagonistic microorganisms.** Neighbor-joining phylogenetic tree constructed with 16S rRNA gene sequences (1390 bp) showing the relationships between strain B-28 (identified as *Sphingomonas aquatilis*) and other related taxa in the genus *Sphingomonas*. The phylogenetic tree was rooted to *Blastomonas aquatica* KJ528316. T indicates type strain.

**Figure S5.** **Metabolomic Quality Assessment.** The total ion current (TIC) of the QC samples in positive ion mode **(A)** and negative ion mode **(B)**, PCA score plot of the total metabolite profiles **(C)**, and Pearson's correlation coefficient analysis of normal (N) and post-herbivory (P) samples **(D).**

**Figure S6. The proportion of different metabolites**

**Figure S7. The visual representation of the results obtained from the differential expression analysis.** Volcano maps of differentially expressed metabolites **(A)** and genes **(C)**. Bar charts of the significant differential metabolites **(B)** and genes **(D)**.

**Figure S8. The lollipop and bar chart diagram of KEGG pathway enrichment analysis of top 30 differentially expressed metabolites (A) and genes (B).**

**Figure S9.** **GO function annotation analysis. (A)** Histogram of GO classification statistics of differentially expressed genes. BP, biological process. CC, cellular component. MF, molecular function. **(B)** The top 20 GO pathways enrichment of differentially expressed genes.

**Figure S10. Metabolic saccharides promote the growth of *S. aquatilis*. (A)** The promoting effects of stachyose, D-sorbitol, and D-mannitol with different concentrations on *S. aquatilis*. **(B)** The utilization efficiency of different carbon sources at a concentration of 5 g/L.

**Figure S11. Absolute abundance of *Sphingomonas* in different samples.** Error bars represent the standard deviations. Different lowercase letters represent significant differences between different groups (*p* < 0.05).
