# Supplementary material for: Multiomics reveals postherbivory tea leaves restructure endophytic microbiota to resist disease and boost saccharide metabolism to promote growth
Source: Hortic Res. 2026 Aug 4;13(8):uhag338. doi: 10.1093/hr/uhag338 (PMC13482628; doi:10.1093/hr/uhag338)
Supplement: Supplementary file 1 [file web_material_uhag338.zip › Supplementary Table.docx]

**Table S1. Pathogen genera with significantly increased relative abundance in different host plants**

| **Potential Pathogen Genus** | **Host** | **Reference** |
| --- | --- | --- |
| ***Pestalotiopsis*** | ***Camellia sinensis*** | 1-3 |
| ***Davidiella*** | *Avena nuda* | 4 |
| ***Alternaria*** | ***Camellia sinensis*** | 5-7 |
| ***Haematonectria*** | *Citrus* | 8 |
| ***Phaeosphaeria*** | *Zea mays* | 9 |
| ***Strelitziana*** | *Malus pumila* | 10 |
| ***Mycosphaerella*** | *Diospyros kaki*, *Musa nana* | 11,12 |

**Table S2. Empirical and randomized molecular ecology network properties of microbial communities from Normal and Post-herbivory leaf samples.**

|  | | Bacteria | | Fungi | |
| --- | --- | --- | --- | --- | --- |
|  |  | Normal | Post-herbivory | Normal | Post-herbivory |
| Empirical networks | Similarity threshold | 0.91 | 0.93 | 0.93 | 0.94 |
|  | Total nodes | 253 | 574 | 61 | 171 |
|  | Total links | 875 | 9676 | 73 | 833 |
|  | The ratio of positive links | 77.83% | 99.58% | 73.97% | 97.72% |
|  | The ratio of negative links | 22.17% | 0.42% | 26.03% | 2.28% |
|  | R^2^ | 0.982 | 0.980 | 0.995 | 0.958 |
|  | Average degree | 6.917 | 33.714 | 2.393 | 9.743 |
|  | Average path distance | 4.048 | 3.340 | 4.082 | 3.666 |
|  | Average clustering coefficient | 0.282 | 0.428 | 0.157 | 0.333 |
|  | Modularity | 0.434 | 0.233 | 0.696 | 0.282 |
| Random networks | Average path distance (Mean±SD) | 3.088±0.045 | 2.545±0.018 | 3.902±0.243 | 2.728±0.037 |
|  | Average clustering coefficient (Mean±SD) | 0.050±0.009 | 0.159±0.006 | 0.006±0.012 | 0.095±0.011 |
|  | Modularity (Mean±SD) | 0.286±0.006 | 0.096±0.004 | 0.606±0.017 | 0.209±0.006 |

**Table S3. Inter-Domain Ecological Networks properties of microbial communities from Normal and Post-herbivory samples.**

|  |  | Normal | Post-herbivory |
| --- | --- | --- | --- |
| Empirical network | Cutoff | 0.7 | 0.7 |
|  | Number of bacterial species | 326 | 590 |
|  | Number of fungal species | 75 | 169 |
|  | Number of links | 1403 | 6934 |
|  | The ratio of positive links | 50.82% | 23.33% |
|  | The ratio of negative links | 49.18% | 76.67% |
|  | Connectance | 0.057 | 0.070 |
|  | Weighted connectance | 0.065 | 0.080 |
|  | Web asymmetry | 0.626 | 0.555 |
|  | Links per species | 3.499 | 9.136 |
|  | Cluster coefficient | 0.040 | 0.047 |
|  | Average node degree | 6.998 | 18.271 |
|  | Linkage density | 26.139 | 60.754 |
|  | Shannon diversity | 7.246 | 8.844 |
|  | Interaction evenness | 0.717 | 0.768 |
|  | Functional complementarity of bacteria | 475.234 | 1236.814 |
|  | Functional complementarity of fungi | 307.837 | 795.029 |
|  | Modularity(fast_greedy) | 0.458 | 0.440 |
| Random network | Number of compartments (Mean±SD) | 1.530±0.688 | 1.570±0.590 |
|  | Weighted nestedness (Mean±SD) | 0.494±0.015 | 0.597±0.008 |
|  | Modularity(fast_greedy) (Mean±SD) | 0.303±0.004 | 0.163±0.005 |

**Table S4. The expression levels of flavonoids in different samples.**

| **Compounds** | **Normal-1** | **Normal-2** | **Normal-3** | **Post-herbivory-1** | **Post-herbivory-2** | **Post-herbivory-3** | **P-value** | **Log2FC** | **Type** |
| --- | --- | --- | --- | --- | --- | --- | --- | --- | --- |
| 6-C-Glucosyl-2-Hydroxynaringenin | 605775.826 | 702258.7175 | 951186.2235 | 264769.5823 | 266008.9983 | 426580.3827 | 0.033094375 | -1.238693383 | down |
| Eriodictyol-7-O-Rutinoside (Eriocitrin) | 149565.1571 | 149339.6758 | 196922.2963 | 72493.44129 | 107696.7179 | 113493.0629 | 0.031601945 | -0.755576363 | down |
| Hesperetin-5-O-glucoside | 22150942.9 | 25804432.73 | 27943230.25 | 16654083.37 | 17992534.93 | 15086377.8 | 0.020105908 | -0.609870039 | down |
| O-MethylNaringenin-8-C-arabinoside | 606192.589 | 902458.9522 | 894205.6349 | 395822.3058 | 483223.462 | 252422.757 | 0.028230495 | -1.086554446 | down |
| Hesperetin-7-O-glucoside | 4869977.535 | 5076638.139 | 5404973.293 | 3440883.842 | 3546879.489 | 2992987.016 | 0.001523285 | -0.621167804 | down |
| Dihydromyricetin (Ampelopsin) | 696029.2268 | 916557.3597 | 970414.6246 | 479434.6384 | 480037.9672 | 648006.0788 | 0.039199689 | -0.684248713 | down |
| 6-C-MethylKaempferol-3-glucoside | 4593766.08 | 5308411.573 | 5555933.897 | 3448717.848 | 2827363.514 | 2885578.064 | 0.005679002 | -0.754683244 | down |
| Chrysoeriol-7-O-glucoside | 491278.6533 | 570611.6036 | 430351.4266 | 333901.2514 | 320829.4282 | 312521.7845 | 0.046953437 | -0.625516809 | down |
| Diosmetin-7-O-galactoside* | 4421980.196 | 5625799.994 | 4594184.279 | 3408058.703 | 3046301.12 | 2828029.871 | 0.026461956 | -0.657540958 | down |
| 5-Hydroxy-6,7,3',4'-tetramethoxyflavone* | 861333.272 | 828862.902 | 792443.4123 | 1352337.228 | 1446801.152 | 1652348.127 | 0.014240226 | 0.842412347 | up |
| Orientin-2''-O-galactoside | 1274592.003 | 1732325.5 | 1137305.273 | 784827.2603 | 608554.6544 | 546589.976 | 0.040631137 | -1.09506581 | down |
| 7-Benzyloxy-5-hydroxy-3',4'-methylenedioxyflavonoid | 1943911.656 | 2900374.836 | 2498439.951 | 7010276.285 | 7543672.473 | 7089326.284 | 0.000417801 | 1.559531065 | up |
| Diosmetin-7-O-glucoside* | 3788404.581 | 4164990.566 | 4200913.335 | 2769843.67 | 2756754.46 | 2079872.531 | 0.00865669 | -0.676168698 | down |
| 5-Hydroxy-6,7,3',4',5'-Pentamethoxyflavone | 24137.28507 | 16251.62276 | 10475.89279 | 44233.07504 | 41954.72237 | 30519.45069 | 0.019571995 | 1.198154627 | up |
| 3,5,6,7-Tetramethoxyflavone | 2254707.294 | 2102808.408 | 1856607.192 | 3852046.969 | 4364206.544 | 4240399.178 | 0.000617256 | 1.003293766 | up |
| 5,6,7,4'-Tetramethoxyflavone | 79742.90678 | 70530.55749 | 68736.08981 | 124767.7344 | 154015.3825 | 132504.4736 | 0.010079582 | 0.909153736 | up |
| Hispidulin-7-O-glucoside(Homoplantaginin)* | 4862465.336 | 5550167.041 | 5390780.348 | 3716736.22 | 3437314.727 | 2585484.871 | 0.011626261 | -0.698311219 | down |
| 5-Hydroxy-3,7,3',4'-tetramethoxyflavone (Retusin)* | 995146.1536 | 878916.1153 | 808069.1642 | 1430909.839 | 1443877.197 | 1613432.581 | 0.00172285 | 0.742763336 | up |
| Isovitexin-2''-O-(6'''-p-coumaroyl)glucoside | 233866.2174 | 208566.0039 | 202355.1399 | 315442.1398 | 316179.9562 | 347424.4862 | 0.001504571 | 0.602554039 | up |
| Vitexin-7-O-glucoside | 3634296.725 | 4317470.574 | 4652219.897 | 2297291.882 | 1672558.382 | 2271285.403 | 0.006239232 | -1.013999716 | down |
| Luteolin-8-C-arabinoside | 422783.2524 | 437694.8883 | 509063.5757 | 367183.629 | 291634.7066 | 217662.9685 | 0.041223821 | -0.643897989 | down |
| Kaempferol-3-O-(6''-O-acetyl)glucoside | 7112106 | 8385458.504 | 9404244.553 | 4269866.514 | 5330401.063 | 5404025.696 | 0.020664384 | -0.730875187 | down |
| Quercetin-3-O-(4''-O-glucosyl)rhamnoside | 8226400.418 | 8197545.235 | 6720118.519 | 3920767.482 | 2140013.833 | 3464203.77 | 0.003456477 | -1.28085349 | down |
| Isorhamnetin-3-O-gallate | 199278.5427 | 130583.8383 | 177181.5009 | 90516.01598 | 101022.347 | 71541.13355 | 0.04092905 | -0.946611796 | down |
| Quercetin-3-O-(2''-O-galloyl)Arabinoside | 33350.23804 | 37053.29483 | 36100.4917 | 108709.4669 | 86488.3656 | 97157.49693 | 0.008976527 | 1.456814943 | up |
| Hesperetin-6-C-glucoside-7-O-glucoside | 110515.1566 | 98547.69324 | 124113.515 | 89332.77158 | 70996.8404 | 54994.40331 | 0.037587032 | -0.629776828 | down |
| kaempferol-3-p-coumaroyldiglucoside-7-glucoside | 126410.0718 | 170767.8079 | 184263.3767 | 115305.4024 | 83288.27327 | 84418.0835 | 0.042042501 | -0.766497778 | down |
| Myricetin-3-O-galactoside* | 757393.3474 | 993132.9195 | 842035.9643 | 473088.8412 | 486949.1925 | 614124.5072 | 0.019766003 | -0.719794105 | down |
| Quercetin-3-O-xyloside (Reynoutrin)* | 898552.1593 | 1362654.743 | 1092154.323 | 346557.6551 | 757925.287 | 567704.1095 | 0.03623588 | -1.003871662 | down |

**Table S5. The expression levels of saccharide metabolites in different samples.**

| **Compounds** | **Normal-1** | **Normal-2** | **Normal-3** | **Post-herbivory-1** | **Post-herbivory-2** | **Post-herbivory-3** | **P-value** | **Log2FC** | **Type** |
| --- | --- | --- | --- | --- | --- | --- | --- | --- | --- |
| **D-Mannitol** | 21027.42 | 22950.77 | 24810.33 | 40278.57 | 33769.89 | 37544.25 | 0.005899 | 0.698003 | up |
| **Stachyose** | 314871.4 | 203293.6 | 217775.3 | 431799.7 | 325586.9 | 452455.4 | 0.040593 | 0.717158 | up |
| **Trehalose 6-phosphate** | 1259398 | 502259.5 | 433578.4 | 3611248 | 3051281 | 2362109 | 0.008881 | 2.039493 | up |
| **D-Maltotetraose** | 68546.76 | 39605.58 | 35330.21 | 86747.05 | 123262.7 | 89372.78 | 0.030402 | 1.061115 | up |
| **D-Sorbitol** | 114425.4 | 136824 | 120357.5 | 213440 | 200604.4 | 184832.9 | 0.002414 | 0.688483 | up |
| **DMelezitose O-rhamnoside** | 28970.07 | 26019.96 | 53220.21 | 76347.78 | 104265.4 | 69439.83 | 0.027822 | 1.208397 | up |

**Table S6. Strains used in the *Alternaria* spp. phylogenetic tree construction and their GenBank accession numbers.**

| **Species name** | **Accession number** | **GenBank accession numbers** | | **Reference** |
| --- | --- | --- | --- | --- |
|  |  | **ITS** | **GAPDH** |  |
| *Alternaria agripestis* | CBS 577.94 | KJ718099 | JQ646356 | 13 |
| *Alternaria allii* | CBS 107.28 | KJ718100 | KJ717954 | 13 |
| *Alternaria alternariacida* | CBS 105.51 | KJ718105 | KJ717959 | 13 |
| *Alternaria azadirachtae* | CBS 116444 | KJ718115 | KJ717967 | 13 |
| *Alternaria bataticola* | CBS 531.63 | KJ718117 | JQ646349 | 13 |
| *Alternaria alternata* | EGS 34-016 | AF347031 | AY278808 | 14 |
| *Alternaria aspera* | CBS 115269 | KC584242 | KC584166 | 15 |
| *Alternaria axiaeriisporifera* | CBS 118715 | KC584184 | KC584101 | 15 |
| *Alternaria avenicola* | CBS 121459 | KC584183 | KC584100 | 15 |
| *Alternaria argyranthemi* | CBS 116530 | KC584181 | KC584098 | 15 |
| *Alternaria armoraciae* | CBS 118702 | KC584182 | KC584099 | 15 |
| *Alternaria anigozanthi* | CBS 121920 | KC584180 | KC584097 | 15 |
| *Bipolaris cynodontis* | CBS 109894 | KJ909767 | KM034838 | 16 |

**Table S7. Primers used in this study**

| Usage Primer | of name | Sequence (5’-3’) |
| --- | --- | --- |
| As ODN | AsODN-α-gal | GGTTTTACTGCATGTTTGAG |
|  | sODN-α-gal | CUCAAACAUGCAGUAAAACC |
|  | AsODN-GSGT1 | TGATTAATCGGTATGGGTCG |
|  | sODN-GSGT1 | CGACCCAUACCGAUUAAUCA |
|  | AsODN-Gols4 | CTTTCAAACTCAATCTCGCT |
|  | sODN-Gols4 | AGCGAGAUUGAGUUUGAAAG |
| qRT-PCR | α-gal-F | GATGGCGATTTGGAGGTTTGG |
|  | α-gal-R | AGCAGTGATGGTAGCTGTCG |
|  | GSGT1-F | CTCTCGGACGTGTAGGTGATG |
|  | GSGT1-R | CATGTGACAACCTTGCAGCC |
|  | Gols4-F | TCCTCCTGGACCCTTTGGAG |
|  | Gols4-R | TAGTTCCATGGTTTTGACCCAGTT |

**Reference**

1. Wang, Y. et al. Diversity of *Pestalotiopsis*-Like Species Causing Gray Blight Disease of Tea Plants (*Camellia sinensis*) in China, Including two Novel *Pestalotiopsis* Species, and Analysis of Their Pathogenicity. *Plant Dis.* **103**, 2548–2558 (2019).
2. Xia, Z. et al. Transcriptome Profiling of the Leaf Spot Pathogen, *Pestalotiopsis trachicarpicola*, and Its Host, Tea (*Camellia sinensis*), During Infection. *Plant Dis.* **106**, 2247–2252 (2022).
3. Yang, Y. et al. Analysis of Competing Endogenous RNAs and MicroRNAs in Tea (*Camellia sinensis*) Leaves During Infection by the Leaf Spot Pathogen *Pestalotiopsis trachicarpicola*. *Mol Plant Microbe In* **35**, 432–438 (2022).
4. Liu, L., Ma, M., Liu, Z., Zhang, L., & Zhou, J. Community structure of fungal pathogens causing spikelet rot disease of naked oat from different ecological regions of China. *Sci. Rep.* **11**, 1243 (2021).
5. Zhou, L. X. & Xu, W. X. First Report of *Alternaria alternata* Causing Leaf Spots of Tea (*Camellia sinensis*) in China. *Plant Dis.* **98**, 697 (2014).
6. Chen, Y. J. et al. Phylogenetic and morphological characteristics of *Alternaria alternata* causing leaf spot disease on *Camellia sinensis* in China. *Australasian Plant Pathol.* **47**, 335–342 (2018).
7. Yin, Q. X. et al. First report of *Alternaria longipes* causing leaf spot on tea in China. *Plant Dis.* **105**, 4167 (2021).
8. Yago, J. I. & Chung, K. R. First Report of Twig Blight Disease of Citrus Caused by *Haematonectria haematococca* in the Philippines. *Plant Dis.* **95**, 1590 (2011).
9. Carson M. L. Inheritance of Resistance to *Phaeosphaeria* Leaf Spot of Maize. *Plant Dis.* **85**, 798–800 (2001).
10. Zhang, R. et al. *Strelitziana mali*, a new species causing sooty blotch on apple fruit. *Mycotaxon* **110**, 477-485 (2009).
11. Churchill A. C. *Mycosphaerella fijiensis*, the black leaf streak pathogen of banana: progress towards understanding pathogen biology and detection, disease development, and the challenges of control. *Mol. Plant Pathol* **12**, 307–328 (2011).
12. Lee, S., Lim, Y. & Jung, H. Y. Molecular Phylogeny and Morphology of *Mycosphaerella nawae*, the Causal Agent of Circular Leaf Spot on Persimmon. *Mycobiology* **44**, 191–201 (2016).
13. Woudenberg, J. H. C. et al. Large-spored *Alternaria* pathogens in section *Porri* disentangled. *Stud. Mycol.* **79**, 1–47 (2014).
14. Woudenberg, J. H. et al. *Alternaria* section *Alternaria*: Species, *formae speciales* or pathotypes?. *Stud. Mycol.* **82**, 1–21 (2015).
15. Woudenberg, J. H. C. et al. *Alternaria* redefined. *Stud. Mycol.* **75**, 171–212 (2013).
16. Manamgoda, D. S. et al. The genus *Bipolaris*. *Stud. Mycol.* **79**, 221–288 (2014).
